# Supplementary material for: Nonlinear feedback drives homeostatic plasticity in H2O2 stress response
Source: eLife. 2017 Apr 18;6:e23971. doi: 10.7554/eLife.23971 (PMC5438251; doi:10.7554/eLife.23971)
Supplement: Supplementary file 1. — Table listing the strains used in this study as well as additional information about the genotypes and the origins of the strains. DOI: http://dx.doi.org/10.7554/eLife.23971.026 [file elife-23971-supp1.docx]

| Name | *Background* | *Relevant Genotype* | *Genotype* | Origin |
| --- | --- | --- | --- | --- |
| BY4742 | *S288C* | *WT* | *MATα; his3∆1; leu2∆0 ; lys2Δ0; met15∆0; ura3∆0* | Euroscarf |
| Y10569 | *S288C (BY4742)* | *yap1Δ* | *BY4742; MATα; ura3Δ0; leu2Δ0; his3Δ1; lys2Δ0; YML007w::kanMX4* | Euroscarf |
| Y12667 | *S288C (BY4742)* | *erg3Δ* | *BY4742; MATα; ura3Δ0; leu2Δ0; his3Δ1; lys2Δ0; YLR056w::kanMX4* | Euroscarf |
| Y10568 | *S288C (BY4742)* | *erg6Δ* | *BY4742; MATα; ura3Δ0; leu2Δ0; his3Δ1; lys2Δ0; YML008c::kanMX4* | Euroscarf |
| Y15982 | *S288C (BY4742)* | *ccp1Δ* | *BY4742; MATα; ura3Δ0; leu2Δ0; his3Δ1; lys2Δ0; YKR066c::kanMX4* | Euroscarf |
| Y14935 | *S288C (BY4742)* | *srx1Δ* | *BY4742; MATα; ura3Δ0; leu2Δ0; his3Δ1; lys2Δ0; YKL086w::kanMX4* | Euroscarf |
| Y14287 | *S288C (BY4742)* | *tsa2Δ* | *BY4742; MATα; ura3Δ0; leu2Δ0; his3Δ1; lys2Δ0; YDR453c::kanMX4* | Euroscarf |
| Y10545 | *S288C (BY4742)* | *tsa1Δ* | *BY4742; MATα; ura3Δ0; leu2Δ0; his3Δ1; lys2Δ0; YML028w::kanMX4* | Euroscarf |
| BY4741  Tsa1-GFP | *S288C (BY4741)* | *TSA1-GFP* | *BY4741; MATa; his3Δ1; leu2Δ0; met15D0; ura3Δ0; TSA1-GFP::His3MX6* | (Huh et al., 2003) |
| BY4741  Rnr3-GFP | *S288C (BY4741)* | *RNR3-GFP* | *BY4741; MATa; his3Δ1; leu2Δ0; met15D0; ura3Δ0; RNR3-GFP::His3MX6* | (Huh et al., 2003) |
| BY4741  Ddc2-GFP | *S288C (BY4741)* | *DDC2-GFP* | *BY4741; MATa; his3Δ1; leu2Δ0; met15D0; ura3Δ0; DDC2-GFP::His3MX6* | (Huh et al., 2003) |
| BY4741  Trx2-GFP | *S288C (BY4741)* | *TRX2-GFP* | *BY4741; MATa; his3Δ1; leu2Δ0; met15D0; ura3Δ0; TRX2-GFP::His3MX6* | (Huh et al., 2003) |
| BY4741  Hsp104-GFP | *S288C (BY4741)* | *HSP104-GFP* | *BY4741; MATa; his3Δ1; leu2Δ0; met15D0; ura3Δ0; HSP104-GFP::His3MX6* | (Huh et al., 2003) |
| YG230 | *S288C (BY4741)* | *YAP1-GFP; HTB2-mCherry* | *BY4741; MATa; his3Δ1; leu2Δ0; met15D0; ura3Δ0; YAP1 -GFP::His3MX6 ; HTB2-mCherry ::kanMX4* | This study |
| YCG02 | *S288C (BY4742)* | *HTB2-sfGFP* | *BY4741; MATa; his3Δ1; leu2Δ0; met15D0; ura3Δ0; HTB2-sfGFP::kanMX4* | This study |
| YG237 | *S288C (BY4741)* | *tsa1Δ; tsa2Δ; ACT1pr::TSA1-GFP* | *BY4741; MATa; his3Δ1; leu2Δ0; met15D0; ura3Δ0; tsa1pΔ::kanMX4-ACT1pr::TSA1-GFP::His3MX6; tsa2Δ::kanMX4* | This study |
| SY992 | *S288C* | *WT* | *MATα; his3∆1; leu2∆0 ; lys2Δ0; met15∆0; ura3∆0; trp1-63; ade2∆0; ADE8* | Euroscarf |
| YG228 | *S288C (SY992)* | *TSA1pr-sfGFP-deg* | *SY992; MATα; his3∆1; leu2∆0 ; lys2Δ0; met15∆0; ura3∆0; trp1-63; TSA1pr-sfGFP-degron::kanMX4* | This study |
| YG220 | *S288C (SY992)* | *TRX2pr-sfGFP-deg* | *SY992; MATα; his3∆1; leu2∆0 ; lys2Δ0; met15∆0; ura3∆0; trp1-63; TRX2pr-sfGFP-degron::kanMX4* | This study |
| Y252 | *S288C* | *WT* | *MATa;* *ura3-52; lys2-801^amber^; ade2-101^ochre^; trp1∆1; leu2∆1* | (Sikorski and Hieter, 1989) |
| Y252  *msn2∆; msn4∆* | *S288C (Y252)* | *msn2∆; msn4∆* | *Y252; MATa;* *ura3-52; lys2-801^amber^; ade2-101^ochre^; trp1∆1; leu2∆1; msn2∆::HIS3 msn4∆::URA3* | C. Godon |
| Y252  *ctt1∆; cta1∆* | *S288C (Y252)* | *ctt1∆; cta1∆* | *Y252; MATa;* *ura3-52; lys2-801^amber^; ade2-101^ochre^; trp1∆1; leu2∆1; ctt1∆::KAN; cta1∆::NAT* | This study |
| Y252  *trx1∆; trx2∆* | *S288C (Y252)* | *trx1∆; trx2∆* | *Y252; MATa;* *ura3-52; lys2-801^amber^; ade2-101^ochre^; trp1∆1; leu2∆1; trx1::URA3; trx2::KAN* | D. Spector |
| BY4741  *tsa1Δ; tsa2Δ* | *S288C (BY4741)* | *tsa1Δ; tsa2Δ* | *BY4741; MATa; his3Δ1; leu2Δ0; met15D0; ura3Δ0;  tsa1Δ::KAN; tsa2Δ::TRP1* | This study |
| BY4741 *tsa1Δ; tsa2Δ; ahp1Δ* | *S288C (BY4741)* | *tsa1Δ; tsa2Δ; ahp1Δ* | *BY4741; MATa; his3Δ1; leu2Δ0; met15D0; ura3Δ0;  tsa1Δ::KanMX4; tsa2Δ::TRP1; ahp1Δ::TRP1* | M. E. Huang |
| BY4742 *tsa1C171S; Δtsa2* | *S288C (BY4742)* | *tsa1C171S; Δtsa2* | *BY4742; MATα; his3∆1; leu2∆0 ; lys2Δ0; met15∆0; ura3∆0 ; tsa1C171S; tsa2Δ::KIURA3* | This study |
| BY4742  *tsa1ΔYF; Δtsa2* | *S288C (BY4742)* | *tsa1ΔYF; Δtsa2* | *BY4742; MATα; his3∆1; leu2∆0 ; lys2Δ0; met15∆0; ura3∆0; tsa1ΔYF; tsa2Δ::KIURA3* | This study |
